# Supplementary material for: Sphingomonas and Phenylobacterium as Major Microbiota in Thymic Epithelial Tumors
Source: J Pers Med. 2021 Oct 26;11(11):1092. doi: 10.3390/jpm11111092 (PMC8623653; doi:10.3390/jpm11111092)
Supplement: Supplementary file 1 [file jpm-11-01092-s001.zip › jpm-1367448-supplementary/jpm-1367448 sup/Supplementary Figure S1.pdf]

Supplementary Figure 1.

No.	Type	Streptococcaceae	Sphingomonadaceae	Bacillales incertae sedis	Bradyrhizobiaceae	Neisseriaceae	Nocardioidaceae	Burkholderiaceae	Staphylococcaceae	Corynebacteriaceae	Methylobacteriaceae	Fusobacteriaceae	Clostridiaceae	unclassified Burkholderiales	Promicromonosporaceae	Clostridiales Family XI. Incertae Sedis	Salinisphaeraceae	Bacillaceae	Bacillales Family X. Incertae Sedis	Rhodocyclaceae	Moraxellaceae	Pasteurellaceae	Phormidiaceae	Saprospiraceae	Xanthomonadaceae	Enterococcaceae	Flavobacteriaceae	Bifidobacteriaceae	Caulobacteraceae	Phyllobacteriaceae	Dietziaceae
1	A		65.7%		3.7%																								30.6%		
2	A		41.2%		2.2%		0.4%			10.4%	1.4%		3.8%	0.3%	1.0%			3.3%	2.7%		0.7%			1.3%		5.6%	12.7%	1.5%	9.6%	2.0%	
3	A		66.2%		6.9%																								23.0%	3.9%	
4	A		67.0%		8.2%				1.0%		1.5%																		21.8%	0.6%	
5	A	2.4%	61.1%		5.8%	0.4%		0.1%	0.6%		0.4%	0.1%								0.2%	0.2%								25.4%	2.6%	0.7%
6	AB_A	0.3%	63.4%	0.3%	6.8%																								27.3%	1.9%	
7	AB_A		35.6%		27.4%						0.3%																		31.5%	5.1%	
8	AB_A		62.1%		8.6%																								26.9%	2.3%	
9	AB_B		100.0 %																												
10	AB_B		50.6%		2.9%													7.0%	1.8%				7.9%			5.3%	1.8%		20.2%	2.6%	
11	AB_B	1.0%	58.4%		5.9%				0.9%	1.2%	2.5%												2.3%		1.1%				22.8%	3.2%	0.7%
12	B1		63.9%		8.6%																								24.5%	3.0%	
13	B1		61.7%		10.0%																								25.6%	2.7%	
14	B1		63.9%		4.8%						2.0%						0.8%												26.4%	2.2%	
15	B1		62.5%		2.8%						0.3%						0.6%												31.4%	2.4%	
16	B1		72.2%		6.7%																								21.1%		
17	B2		68.4%		5.3%						0.7%					0.6%	0.7%				1.5%	1.1%							19.7%	2.0%	
18	B2		60.2%		7.1%						0.8%																		28.7%	3.1%	
19	B2		69.8%		5.3%																								23.4%	1.5%	
20	B2		54.5%		4.8%					18.5%	1.4%																		20.8%		
21	B3		54.4%		9.9%						0.4%										0.8%								34.2%	0.4%	
22	B3		61.0%		4.1%						0.8%																		31.5%	2.6%	
MEAN		0.2%	62.0%	0.0%	6.7%	0.0%	0.0%	0.0%	0.1%	1.4%	0.6%	0.0%	0.2%	0.0%	0.0%	0.0%	0.1%	0.5%	0.2%	0.0%	0.1%	0.0%	0.5%	0.1%	0.1%	0.5%	0.7%	0.1%	23.9%	2.0%	0.1%
±SD		0.5%	12.2%	0.1%	5.3%	0.1%	0.1%	0.0%	0.3%	4.4%	0.7%	0.0%	0.8%	0.1%	0.2%	0.1%	0.2%	1.6%	0.7%	0.0%	0.4%	0.2%	1.7%	0.3%	0.2%	1.6%	2.7%	0.3%	7.6%	1.4%	0.2%

Composition and abundance of dominant families in all samples. The abundance values of the detected families are presented using a heatmap.
