# Supplementary material for: Sphingomonas and Phenylobacterium as Major Microbiota in Thymic Epithelial Tumors
Source: J Pers Med. 2021 Oct 26;11(11):1092. doi: 10.3390/jpm11111092 (PMC8623653; doi:10.3390/jpm11111092)
Supplement: Supplementary file 1 [file jpm-11-01092-s001.zip › jpm-1367448-supplementary/jpm-1367448 sup/Supplementary Figure S2.pdf]

Supplementary Figure 2.

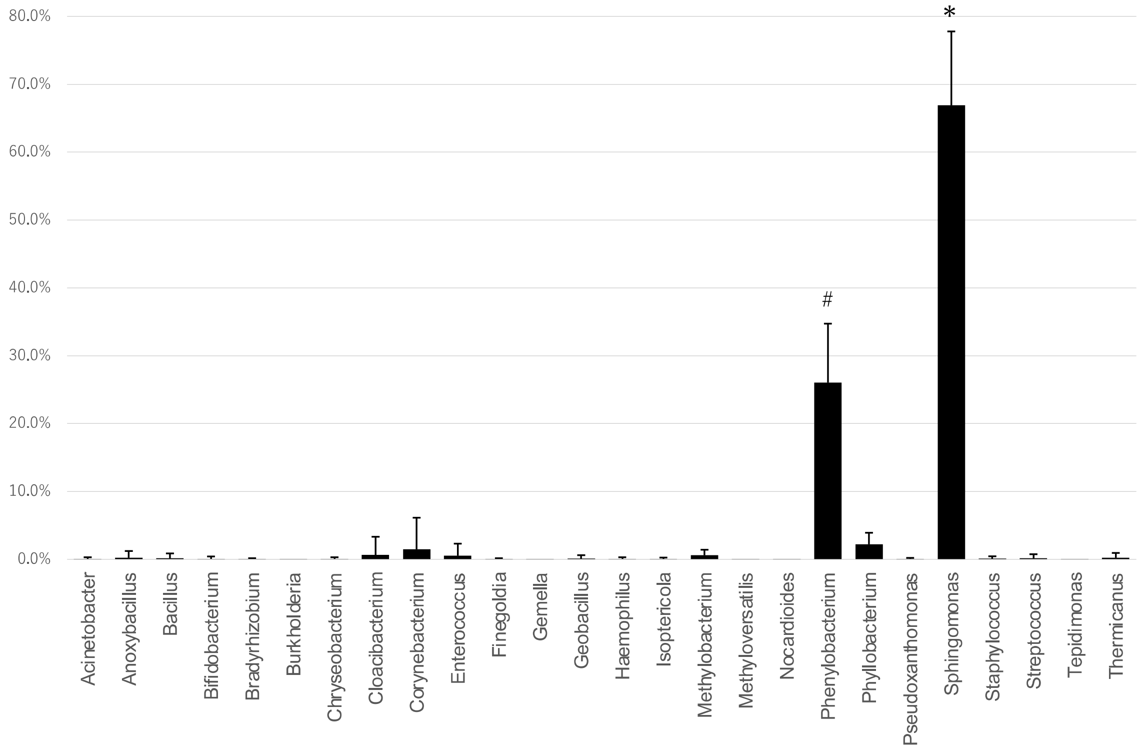

Comparison of abundance of detected genera. *Sphingomonas* and *Phenylobacterium* were significantly more abundant than the others. *$p<0.05$ compared to the other genera, #$p<0.05$ compared to the other genera except *Sphingomonas*.
