# Supplementary material for: Sphingomonas and Phenylobacterium as Major Microbiota in Thymic Epithelial Tumors
Source: J Pers Med. 2021 Oct 26;11(11):1092. doi: 10.3390/jpm11111092 (PMC8623653; doi:10.3390/jpm11111092)
Supplement: Supplementary file 1 [file jpm-11-01092-s001.zip › jpm-1367448-supplementary/jpm-1367448 sup/Supplementary Figure S3.pdf]

Supplementary Figure 3.

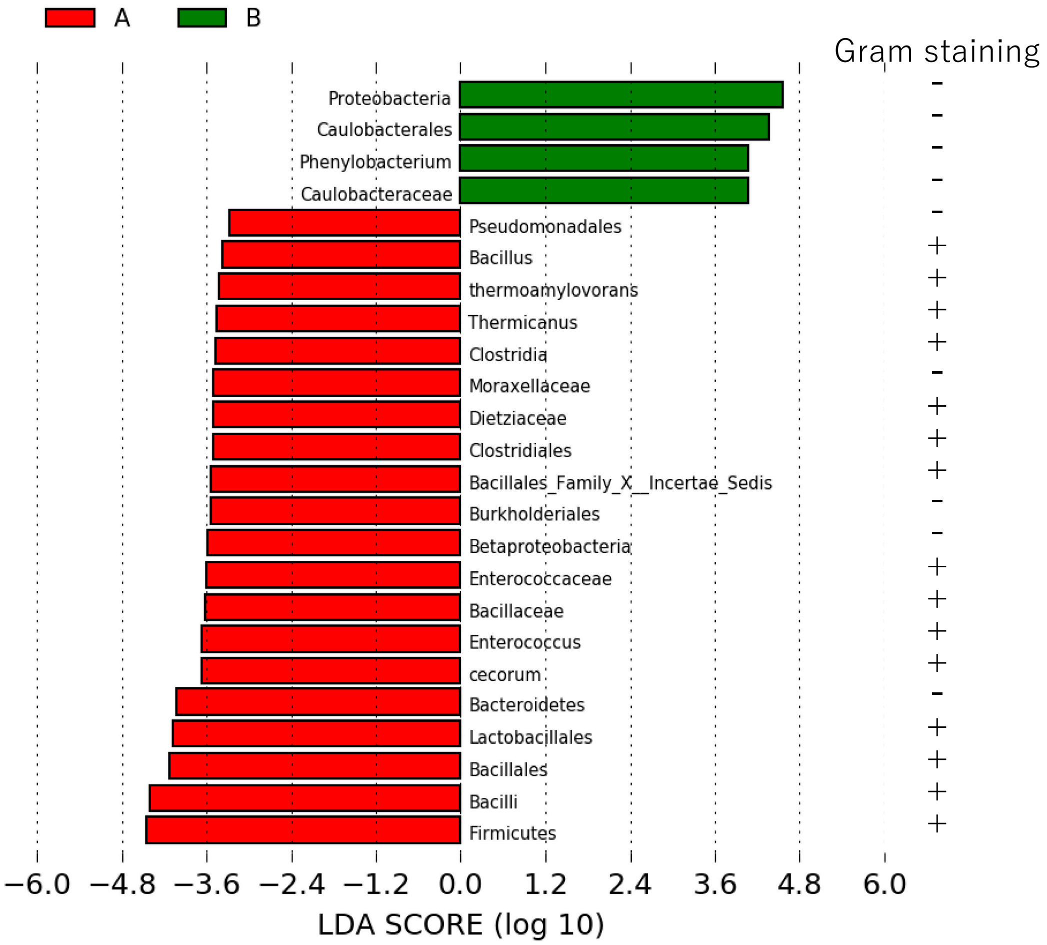

LEfSe analysis between Type A and B histology. This figure presents taxa detected when the *P*-value from the Kruskal-Wallis test for relative abundance between type A and B histology groups was increased from 0.05 to 0.1. Dominant taxa are indicated in red for Type A histology and in green for Type B histology.
