# Supplementary material for: Sphingomonas and Phenylobacterium as Major Microbiota in Thymic Epithelial Tumors
Source: J Pers Med. 2021 Oct 26;11(11):1092. doi: 10.3390/jpm11111092 (PMC8623653; doi:10.3390/jpm11111092)
Supplement: Supplementary file 1 [file jpm-11-01092-s001.zip › jpm-1367448-supplementary/jpm-1367448 sup/Supplementary Table 1.pdf]

Supplementary Table 1. Characteristics of the patients with pancreatic cancer.

Parameter		Number of patients	Overall percentage
Total number		30	
Age, median (range)		74 (49–86)	
Sex			
	Male	13	43.3%
	Female	17	56.7%
Stage			
	I	6	20.0%
	II	12	40.0%
	III	3	10.0%
	IV	9	30.0%
Smoking Status (Pack year)			
	0	15	50.0%
	0<PY≤30	9	30.0%
	>30	6	20.0%
Pathology			
	IDC (Adenocarcinoma)	29	96.7%
	IDC (Mucinous carcinoma)	1	3.3%

IDC, invasive ductal carcinoma.
