# Supplementary material for: Sphingomonas and Phenylobacterium as Major Microbiota in Thymic Epithelial Tumors
Source: J Pers Med. 2021 Oct 26;11(11):1092. doi: 10.3390/jpm11111092 (PMC8623653; doi:10.3390/jpm11111092)
Supplement: Supplementary file 1 [file jpm-11-01092-s001.zip › jpm-1367448-supplementary/jpm-1367448 sup/Supplementary Table S2.pdf]

Supplementary Table 2. Read depth of the sequencing.

Case No.	Phylum	Class	Order	Family	Genus	Species	Read_depth
1	Actinobacteria	Actinobacteria	Actinomycetales	Corynebacteriaceae	Corynebacterium	(slash calls)	359
1	Actinobacteria	Actinobacteria	Actinomycetales	Nocardioidaceae	Nocardioides	(genus level ID only)	13
1	Actinobacteria	Actinobacteria	Actinomycetales	Promicromonosporaceae	Isoptericola	(genus level ID only)	34
1	Actinobacteria	Actinobacteria	Bifidobacteriales	Bifidobacteriaceae	Bifidobacterium	(slash calls)	52
1	Bacteroidetes	Flavobacteriia	Flavobacteriales	Flavobacteriaceae	Chryseobacterium	(genus level ID only)	38
1	Bacteroidetes	Flavobacteriia	Flavobacteriales	Flavobacteriaceae	Cloacibacterium	(slash calls)	399
1	Bacteroidetes	Sphingobacteriia	Sphingobacteriales	Saprospiraceae	(family level ID only)		44
1	Firmicutes	Bacilli	Bacillales	Bacillaceae	Bacillus	(slash calls)	17
1	Firmicutes	Bacilli	Bacillales	Bacillaceae	Bacillus	thermoamylovorans	22
1	Firmicutes	Bacilli	Bacillales	Bacillaceae	Geobacillus	stearothermophilus	74
1	Firmicutes	Bacilli	Bacillales	Bacillales Family X	Thermicanus	(genus level ID only)	92
1	Firmicutes	Bacilli	Lactobacillales	Enterococcaceae	Enterococcus	cecorum	194
1	Firmicutes	Clostridia	Clostridiales	Clostridiaceae	(slash calls)		131
1	Proteobacteria	Alphaproteobacteria	Caulobacterales	Caulobacteraceae	Phenylobacterium	(genus level ID only)	331
1	Proteobacteria	Alphaproteobacteria	Rhizobiales	Bradyrhizobiaceae	(slash calls)		76
1	Proteobacteria	Alphaproteobacteria	Rhizobiales	Methylobacteriaceae	Methylobacterium	(slash calls)	49
1	Proteobacteria	Alphaproteobacteria	Rhizobiales	Phyllobacteriaceae	Phyllobacterium	(slash calls)	69
1	Proteobacteria	Alphaproteobacteria	Sphingomonadales	Sphingomonadaceae	Sphingomonas	(slash calls)	1420
1	Proteobacteria	Betaproteobacteria	Burkholderiales	unclassified Burkholderiales	Tepidimonas	fonticaldi	11
1	Proteobacteria	Gammaproteobacteria	Pseudomonadales	Moraxellaceae	Acinetobacter	radioresistens	24
2	Bacteroidetes	Flavobacteriia	Flavobacteriales	Flavobacteriaceae	Cloacibacterium	(slash calls)	12
2	Cyanobacteria	Oscillatoriophycideae	Oscillatoriales	Phormidiaceae	(family level ID only)		52
2	Firmicutes	Bacilli	Bacillales	Bacillaceae	Anoxybacillus	(slash calls)	28
2	Firmicutes	Bacilli	Bacillales	Bacillaceae	Bacillus	thermoamylovorans	18
2	Firmicutes	Bacilli	Bacillales	Bacillales Family X	Thermicanus	(genus level ID only)	12
2	Firmicutes	Bacilli	Lactobacillales	Enterococcaceae	Enterococcus	cecorum	35
2	Proteobacteria	Alphaproteobacteria	Caulobacterales	Caulobacteraceae	Phenylobacterium	(genus level ID only)	133
2	Proteobacteria	Alphaproteobacteria	Rhizobiales	Bradyrhizobiaceae	(slash calls)		19
2	Proteobacteria	Alphaproteobacteria	Rhizobiales	Phyllobacteriaceae	Phyllobacterium	(slash calls)	17
2	Proteobacteria	Alphaproteobacteria	Sphingomonadales	Sphingomonadaceae	Sphingomonas	(slash calls)	334
3	Actinobacteria	Actinobacteria	Actinomycetales	Corynebacteriaceae	Corynebacterium	(slash calls)	10
3	Actinobacteria	Actinobacteria	Actinomycetales	Corynebacteriaceae	Corynebacterium	jeikeium	30
3	Actinobacteria	Actinobacteria	Actinomycetales	Corynebacteriaceae	Corynebacterium	tuberculostearicum	10
3	Actinobacteria	Actinobacteria	Actinomycetales	Dietziaceae	(family level ID only)		31
3	Cyanobacteria	Oscillatoriophycideae	Oscillatoriales	Phormidiaceae	(family level ID only)		95
3	Firmicutes	Bacilli	Bacillales	Staphylococcaceae	Staphylococcus	(slash calls)	37
3	Firmicutes	Bacilli	Lactobacillales	Streptococcaceae	Streptococcus	(slash calls)	42
3	Proteobacteria	Alphaproteobacteria	Caulobacterales	Caulobacteraceae	(family level ID only)		36
3	Proteobacteria	Alphaproteobacteria	Caulobacterales	Caulobacteraceae	Phenylobacterium	(genus level ID only)	910
3	Proteobacteria	Alphaproteobacteria	Rhizobiales	Bradyrhizobiaceae	(slash calls)		243
3	Proteobacteria	Alphaproteobacteria	Rhizobiales	Methylobacteriaceae	Methylobacterium	(slash calls)	104
3	Proteobacteria	Alphaproteobacteria	Rhizobiales	Phyllobacteriaceae	Phyllobacterium	(slash calls)	134
3	Proteobacteria	Alphaproteobacteria	Sphingomonadales	Sphingomonadaceae	Sphingomonas	(slash calls)	2422
3	Proteobacteria	Gammaproteobacteria	Xanthomonadales	Xanthomonadaceae	(slash calls)		14
3	Proteobacteria	Gammaproteobacteria	Xanthomonadales	Xanthomonadaceae	Pseudoxanthomonas	sp.	32
4	Proteobacteria	Alphaproteobacteria	Caulobacterales	Caulobacteraceae	Phenylobacterium	(genus level ID only)	175
4	Proteobacteria	Alphaproteobacteria	Rhizobiales	Bradyrhizobiaceae	(slash calls)		40
4	Proteobacteria	Alphaproteobacteria	Rhizobiales	Phyllobacteriaceae	Phyllobacterium	(slash calls)	11
4	Proteobacteria	Alphaproteobacteria	Sphingomonadales	Sphingomonadaceae	Sphingomonas	(slash calls)	523
5	Firmicutes	Clostridia	Clostridiales	Clostridiales Family XI	Finegoldia	magna	10
5	Proteobacteria	Alphaproteobacteria	Caulobacterales	Caulobacteraceae	Phenylobacterium	(genus level ID only)	345
5	Proteobacteria	Alphaproteobacteria	Rhizobiales	Bradyrhizobiaceae	(slash calls)		92
5	Proteobacteria	Alphaproteobacteria	Rhizobiales	Methylobacteriaceae	Methylobacterium	(slash calls)	12
5	Proteobacteria	Alphaproteobacteria	Rhizobiales	Phyllobacteriaceae	Phyllobacterium	(slash calls)	35
5	Proteobacteria	Alphaproteobacteria	Sphingomonadales	Sphingomonadaceae	Sphingomonas	(slash calls)	1197
5	Proteobacteria	Gammaproteobacteria	Pasteurellales	Pasteurellaceae	Haemophilus	parainfluenzae	19
5	Proteobacteria	Gammaproteobacteria	Pseudomonadales	Moraxellaceae	(slash calls)		27
5	Proteobacteria	Gammaproteobacteria	Salinisphaerales	Salinisphaeraceae	(family level ID only)		13
6	Proteobacteria	Alphaproteobacteria	Caulobacterales	Caulobacteraceae	Phenylobacterium	(genus level ID only)	113
6	Proteobacteria	Alphaproteobacteria	Rhizobiales	Bradyrhizobiaceae	(slash calls)		34
6	Proteobacteria	Alphaproteobacteria	Rhizobiales	Phyllobacteriaceae	Phyllobacterium	(slash calls)	19
6	Proteobacteria	Alphaproteobacteria	Sphingomonadales	Sphingomonadaceae	Sphingomonas	(slash calls)	325
7	Actinobacteria	Actinobacteria	Actinomycetales	Dietziaceae	(family level ID only)		63
7	Firmicutes	Bacilli	Bacillales	Staphylococcaceae	Staphylococcus	(slash calls)	49
7	Firmicutes	Bacilli	Lactobacillales	Streptococcaceae	Streptococcus	(slash calls)	206
7	Fusobacteria	Fusobacteriia	Fusobacteriales	Fusobacteriaceae	(slash calls)		13
7	Proteobacteria	Alphaproteobacteria	Caulobacterales	Caulobacteraceae	(family level ID only)		79
7	Proteobacteria	Alphaproteobacteria	Caulobacterales	Caulobacteraceae	Phenylobacterium	(genus level ID only)	2133
7	Proteobacteria	Alphaproteobacteria	Rhizobiales	Bradyrhizobiaceae	(slash calls)		501
7	Proteobacteria	Alphaproteobacteria	Rhizobiales	Methylobacteriaceae	Methylobacterium	(slash calls)	38
7	Proteobacteria	Alphaproteobacteria	Rhizobiales	Phyllobacteriaceae	Phyllobacterium	(slash calls)	229
7	Proteobacteria	Alphaproteobacteria	Sphingomonadales	Sphingomonadaceae	Sphingomonas	(slash calls)	5313
7	Proteobacteria	Betaproteobacteria	Burkholderiales	Burkholderiaceae	Burkholderia	multivorans	12
7	Proteobacteria	Betaproteobacteria	Neisseriales	Neisseriaceae	(family level ID only)		33
7	Proteobacteria	Betaproteobacteria	Rhodocyclales	Rhodocyclaceae	Methyloversatilis	universalis	17
7	Proteobacteria	Gammaproteobacteria	Pseudomonadales	Moraxellaceae	Acinetobacter	ursingii	14

8	Proteobacteria	Alphaproteobacteria	Caulobacterales	Caulobacteraceae	Phenylobacterium	(genus level ID only)	423
8	Proteobacteria	Alphaproteobacteria	Rhizobiales	Bradyrhizobiaceae	(slash calls)		105
8	Proteobacteria	Alphaproteobacteria	Rhizobiales	Methylobacteriaceae	Methylobacterium	(slash calls)	12
8	Proteobacteria	Alphaproteobacteria	Rhizobiales	Phyllobacteriaceae	Phyllobacterium	(slash calls)	46
8	Proteobacteria	Alphaproteobacteria	Sphingomonadales	Sphingomonadaceae	Sphingomonas	(slash calls)	886
9	Proteobacteria	Alphaproteobacteria	Caulobacterales	Caulobacteraceae	Phenylobacterium	(genus level ID only)	354
9	Proteobacteria	Alphaproteobacteria	Rhizobiales	Bradyrhizobiaceae	(slash calls)		138
9	Proteobacteria	Alphaproteobacteria	Rhizobiales	Phyllobacteriaceae	Phyllobacterium	(slash calls)	38
9	Proteobacteria	Alphaproteobacteria	Sphingomonadales	Sphingomonadaceae	Sphingomonas	(slash calls)	855
10	Proteobacteria	Alphaproteobacteria	Caulobacterales	Caulobacteraceae	(family level ID only)		20
10	Proteobacteria	Alphaproteobacteria	Caulobacterales	Caulobacteraceae	Phenylobacterium	(genus level ID only)	1212
10	Proteobacteria	Alphaproteobacteria	Rhizobiales	Bradyrhizobiaceae	(slash calls)		109
10	Proteobacteria	Alphaproteobacteria	Rhizobiales	Methylobacteriaceae	Methylobacterium	(slash calls)	11
10	Proteobacteria	Alphaproteobacteria	Rhizobiales	Phyllobacteriaceae	Phyllobacterium	(slash calls)	96
10	Proteobacteria	Alphaproteobacteria	Sphingomonadales	Sphingomonadaceae	Sphingomonas	(slash calls)	2450
10	Proteobacteria	Gammaproteobacteria	Salinisphaerales	Salinisphaeraceae	(family level ID only)		23
11	Proteobacteria	Alphaproteobacteria	Caulobacterales	Caulobacteraceae	(family level ID only)		16
11	Proteobacteria	Alphaproteobacteria	Caulobacterales	Caulobacteraceae	Phenylobacterium	(genus level ID only)	938
11	Proteobacteria	Alphaproteobacteria	Rhizobiales	Bradyrhizobiaceae	(slash calls)		275
11	Proteobacteria	Alphaproteobacteria	Rhizobiales	Methylobacteriaceae	Methylobacterium	(slash calls)	11
11	Proteobacteria	Alphaproteobacteria	Rhizobiales	Phyllobacteriaceae	Phyllobacterium	(slash calls)	10
11	Proteobacteria	Alphaproteobacteria	Sphingomonadales	Sphingomonadaceae	Sphingomonas	(slash calls)	1519
11	Proteobacteria	Gammaproteobacteria	Pseudomonadales	Moraxellaceae	Acinetobacter	ursingii	21
12	Firmicutes	Bacilli	Bacillales	Staphylococcaceae	Staphylococcus	(slash calls)	22
12	Proteobacteria	Alphaproteobacteria	Caulobacterales	Caulobacteraceae	Phenylobacterium	(genus level ID only)	496
12	Proteobacteria	Alphaproteobacteria	Rhizobiales	Bradyrhizobiaceae	(slash calls)		186
12	Proteobacteria	Alphaproteobacteria	Rhizobiales	Methylobacteriaceae	Methylobacterium	(slash calls)	34
12	Proteobacteria	Alphaproteobacteria	Rhizobiales	Phyllobacteriaceae	Phyllobacterium	(slash calls)	13
12	Proteobacteria	Alphaproteobacteria	Sphingomonadales	Sphingomonadaceae	Sphingomonas	(slash calls)	1528
13	Proteobacteria	Alphaproteobacteria	Caulobacterales	Caulobacteraceae	Phenylobacterium	(genus level ID only)	972
13	Proteobacteria	Alphaproteobacteria	Rhizobiales	Bradyrhizobiaceae	(slash calls)		831
13	Proteobacteria	Alphaproteobacteria	Rhizobiales	Bradyrhizobiaceae	Bradyrhizobium	cytisi	15
13	Proteobacteria	Alphaproteobacteria	Rhizobiales	Methylobacteriaceae	Methylobacterium	(slash calls)	10
13	Proteobacteria	Alphaproteobacteria	Rhizobiales	Phyllobacteriaceae	Phyllobacterium	(slash calls)	156
13	Proteobacteria	Alphaproteobacteria	Sphingomonadales	Sphingomonadaceae	Sphingomonas	(slash calls)	1099
14	Firmicutes	Bacilli	Bacillales	Bacillales incertae sedis	Gemella	(slash calls)	12
14	Firmicutes	Bacilli	Lactobacillales	Streptococcaceae	Streptococcus	(slash calls)	11
14	Proteobacteria	Alphaproteobacteria	Caulobacterales	Caulobacteraceae	(family level ID only)		19
14	Proteobacteria	Alphaproteobacteria	Caulobacterales	Caulobacteraceae	Phenylobacterium	(genus level ID only)	1071
14	Proteobacteria	Alphaproteobacteria	Rhizobiales	Bradyrhizobiaceae	(slash calls)		273
14	Proteobacteria	Alphaproteobacteria	Rhizobiales	Phyllobacteriaceae	Phyllobacterium	(slash calls)	77
14	Proteobacteria	Alphaproteobacteria	Sphingomonadales	Sphingomonadaceae	Sphingomonas	(slash calls)	2531
15	Proteobacteria	Alphaproteobacteria	Caulobacterales	Caulobacteraceae	Phenylobacterium	(genus level ID only)	757
15	Proteobacteria	Alphaproteobacteria	Rhizobiales	Bradyrhizobiaceae	(slash calls)		138
15	Proteobacteria	Alphaproteobacteria	Rhizobiales	Methylobacteriaceae	Methylobacterium	(slash calls)	56
15	Proteobacteria	Alphaproteobacteria	Rhizobiales	Phyllobacteriaceae	Phyllobacterium	(slash calls)	64
15	Proteobacteria	Alphaproteobacteria	Sphingomonadales	Sphingomonadaceae	Sphingomonas	(slash calls)	1832
15	Proteobacteria	Gammaproteobacteria	Salinisphaerales	Salinisphaeraceae	(family level ID only)		22
16	Proteobacteria	Alphaproteobacteria	Caulobacterales	Caulobacteraceae	Phenylobacterium	(genus level ID only)	510
16	Proteobacteria	Alphaproteobacteria	Rhizobiales	Bradyrhizobiaceae	(slash calls)		179
16	Proteobacteria	Alphaproteobacteria	Rhizobiales	Phyllobacteriaceae	Phyllobacterium	(slash calls)	62
16	Proteobacteria	Alphaproteobacteria	Sphingomonadales	Sphingomonadaceae	Sphingomonas	(slash calls)	1328
17	Proteobacteria	Alphaproteobacteria	Caulobacterales	Caulobacteraceae	Phenylobacterium	(genus level ID only)	172
17	Proteobacteria	Alphaproteobacteria	Rhizobiales	Bradyrhizobiaceae	(slash calls)		21
17	Proteobacteria	Alphaproteobacteria	Sphingomonadales	Sphingomonadaceae	Sphingomonas	(slash calls)	369
18	Proteobacteria	Alphaproteobacteria	Sphingomonadales	Sphingomonadaceae	Sphingomonas	(slash calls)	31
19	Proteobacteria	Alphaproteobacteria	Caulobacterales	Caulobacteraceae	Phenylobacterium	(genus level ID only)	69
19	Proteobacteria	Alphaproteobacteria	Rhizobiales	Bradyrhizobiaceae	(slash calls)		22
19	Proteobacteria	Alphaproteobacteria	Sphingomonadales	Sphingomonadaceae	Sphingomonas	(slash calls)	236
20	Proteobacteria	Alphaproteobacteria	Caulobacterales	Caulobacteraceae	(family level ID only)		18
20	Proteobacteria	Alphaproteobacteria	Caulobacterales	Caulobacteraceae	Phenylobacterium	(genus level ID only)	921
20	Proteobacteria	Alphaproteobacteria	Rhizobiales	Bradyrhizobiaceae	(slash calls)		122
20	Proteobacteria	Alphaproteobacteria	Rhizobiales	Methylobacteriaceae	Methylobacterium	(slash calls)	25
20	Proteobacteria	Alphaproteobacteria	Rhizobiales	Phyllobacteriaceae	Phyllobacterium	(slash calls)	77
20	Proteobacteria	Alphaproteobacteria	Sphingomonadales	Sphingomonadaceae	Sphingomonas	(slash calls)	1818
21	Proteobacteria	Alphaproteobacteria	Caulobacterales	Caulobacteraceae	Phenylobacterium	(genus level ID only)	231
21	Proteobacteria	Alphaproteobacteria	Rhizobiales	Bradyrhizobiaceae	(slash calls)		74
21	Proteobacteria	Alphaproteobacteria	Rhizobiales	Phyllobacteriaceae	Phyllobacterium	(slash calls)	20
21	Proteobacteria	Alphaproteobacteria	Sphingomonadales	Sphingomonadaceae	Sphingomonas	(slash calls)	533
22	Actinobacteria	Actinobacteria	Actinomycetales	Corynebacteriaceae	Corynebacterium	imitans	149
22	Proteobacteria	Alphaproteobacteria	Caulobacterales	Caulobacteraceae	Phenylobacterium	(genus level ID only)	168
22	Proteobacteria	Alphaproteobacteria	Rhizobiales	Bradyrhizobiaceae	(slash calls)		39
22	Proteobacteria	Alphaproteobacteria	Rhizobiales	Methylobacteriaceae	Methylobacterium	(slash calls)	11
22	Proteobacteria	Alphaproteobacteria	Sphingomonadales	Sphingomonadaceae	Sphingomonas	(slash calls)	439
